# Harnessing meta-analysis and artificial intelligence to reveal conserved regulatory biosignatures of abiotic stress in soybean

**DOI:** 10.1186/s13062-026-00788-2

**Published:** 2026-05-11

**Authors:** Parbej Laskar, Brijendra Singh

**Affiliations:** 1 School of Biosciences and Technology, Vellore Institute of Technology, Vellore, Tamil Nadu India; 2 School of Computer Science Engineering and Information Systems, Vellore Institute of Technology, Vellore, India

**Keywords:** Soybean, Transcriptome, Meta-analysis, Abiotic stress, Gene duplication, Orthologs, WGCNA, Feature selection, Machine learning, Deep learning

## Abstract

**Background:**

Soybeans are widely cultivated worldwide as an important source of edible vegetable oil and protein. Due to climate change, it is repeatedly exposed to various abiotic stressors in its natural habitat. Abiotic stresses such as heat, drought, and salinity severely restrict soybean productivity, yet the conserved molecular mechanisms underlying multi-stress tolerance remain poorly understood. The integrated application of machine learning and co-expression network analysis for robust biosignature and hub gene discovery remains limited. Therefore, this study aimed to identify conserved stress-responsive biosignatures and explore their evolutionary and regulatory significance.

**Results:**

Here, we explored the transcriptional regulation of soybean under multiple abiotic stress conditions, including heat, drought, and salt. A total of 14,503 genes are differentially expressed across three stress conditions, with 466 genes common to all three. Gene Ontology and KEGG pathway analyses indicated that the meta-DEGs primarily participate in oxidative stress, hormone signaling, and metabolic pathways. Segmental duplication is the key driving force of stress response gene expansion, and most of these expansions occurred through the recent whole-genome duplication (WGD) in soybean. The 12 abiotic stress-responsive biosignatures were identified using a wedge co-expression network and machine learning (ML)- based hub genes. A deep neural network (DNN) model was constructed to validate stress biosignatures, achieving 97.39% and 76.47% prediction accuracies on the test and external validation sets, respectively.

**Conclusions:**

Our findings revealed conserved stress-responsive genes, key regulatory hubs, and oxidative stress as a central molecular feature governing multi-stress adaptation. The integration of artificial intelligence enabled accurate validation of biosignatures, offering valuable insights into functional genomics and genomic-assisted breeding strategies. This study offers a strong foundation for AI applications in plant breeding and supplies valuable resources for soybean genetic improvement.

**Graphical Abstract:**

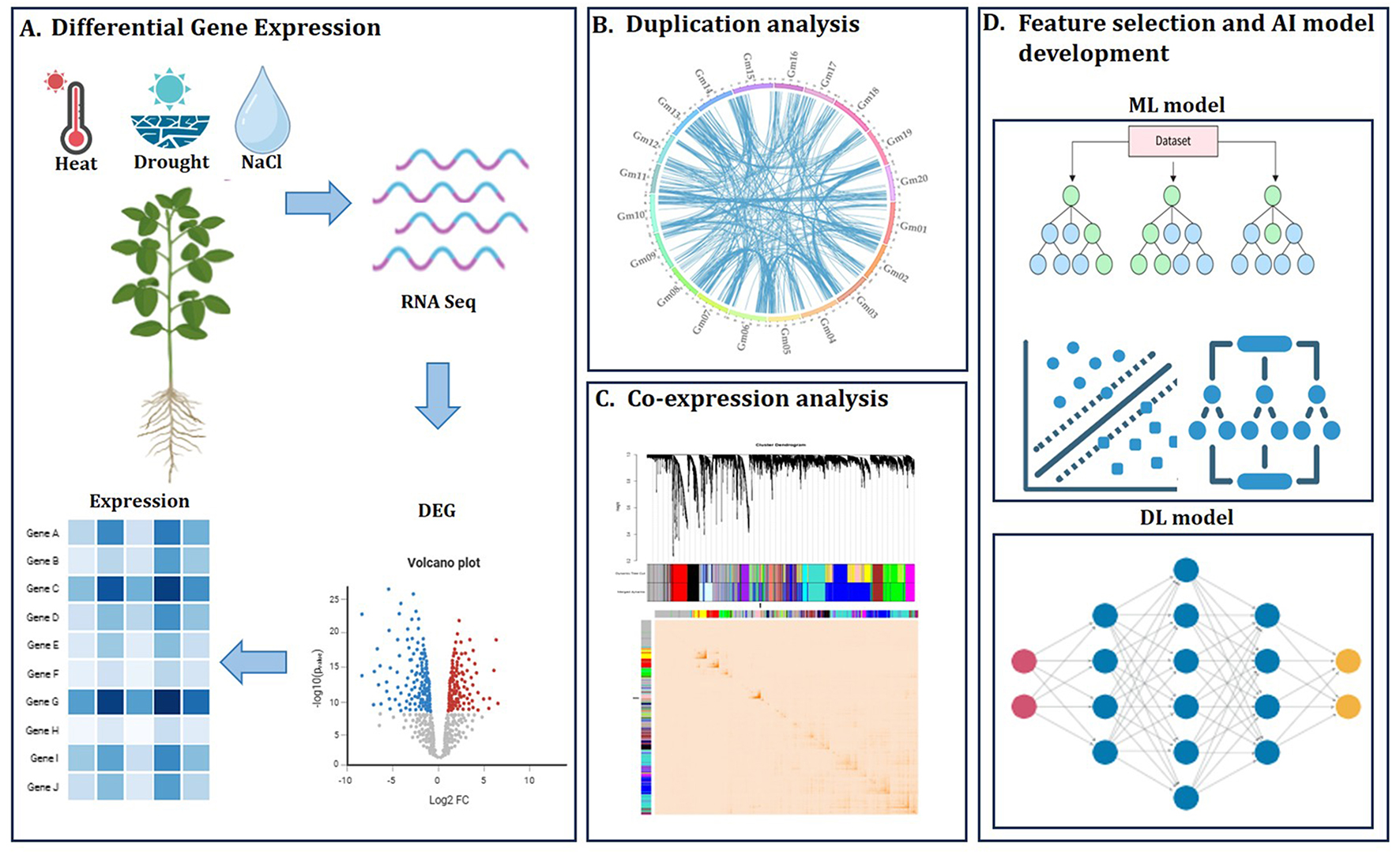

**Supplementary Information:**

The online version contains supplementary material available at 10.1186/s13062-026-00788-2.

## Introduction

The growing population demands more food to sustain itself. By 2050, the world’s human population is projected to reach 9.7 billion. For this instance, to meet the food demand, the production of the food crop must be increased by 70% [1–3]. Unfavourable environmental conditions are a key factor in the failure of major crops to meet demand [4]. Climate change, driven by global warming, leads to extreme environmental conditions, such as increasing temperatures, which in turn intensify drought and soil salinity, ultimately reducing crop yields [5, 6].

*Glycine max* [L.] Merr. (soybean) has been widely cultivated worldwide for its nutritious seeds and is considered a major source of plant protein. Additionally, as a legume, this crop helps restore soil nutrients through biological N2 fixation and reduces dependence on synthetic fertilizers, making it beneficial in crop rotation systems [7]. Soybeans are the most important legume crop cultivated worldwide, including South America, North America, China, and India [8]. Currently, the entire soybean cultivation area is experiencing significant economic losses due to abiotic factors, such as heat, drought, and salinity, resulting in a substantial decline in both yield quantity and quality [9]. Plants are usually subjected to multiple stresses simultaneously in the natural habitat, leading to complex interactions in which one stress can intensify the effects of others. Understanding the perception and integration of multiple abiotic stress signals in soybean has become extremely important for multi-stress tolerance soybean breeding [10, 11]. Transcription factors (TFs) play important role in plant stress responses [12]. Multiple TF families like WRKY, bHLH, and NAC contribute to stress tolerance by controlling the expression of various stress genes, creating a complex co-expression network [13]. Therefore, identifying and characterizing the co-expressed genes in soybean provides valuable targets for genetic approaches to improve stress tolerance.

Omics technologies enable the identification of genes and biological pathways that control plant stress responses. Analyzing transcriptomes has enlightened the identification of gene expression dynamics under stress conditions [14]. Transcriptome analysis has been successfully applied to soybean abiotic stress to identify genes that help plants cope with these conditions. Multiple studies have identified key soybean genes that are differentially expressed under various stresses, including drought [15–17], salt [18, 19], heat [20], combined heat and water deficit [21], as well as alkaline [22] and heavy metal stresses [23, 24].

Meta-analysis of transcriptomic data has garnered greater attention recently, as it allows for the combination of expression datasets and offers a robust method to identify biological phenomena. Additionally, it has gained recognition for its ability to improve statistical power and enhance the generalizability of research findings [25]. Single stress transcriptomic analysis can yield biased or misleading results due to variations in experimental parameters such as treatment type, stress severity, treatment duration, tissue type, plant age, and sample size. The presence of diverse genetic backgrounds in plants also limits the ability of single-stress experiments to fully capture the complexity of plant stress responses, as differences among genotypes lead to genotype-specific variability in responses. A meta-analysis of multiple datasets has been proposed as a way to address these issues, providing a solid foundation for identifying core or hub genes [26, 27]. Additionally, applying artificial intelligence, such as ML models, to large transcriptomic datasets can uncover more universal patterns, especially by addressing batch effects associated with categorical variables [28, 29]. The combination of meta-analysis with an ML-based feature selection method highlights precise identification of the stress-responsive hub genes or biosignatures in some plant species, including *Arabidopsis* [30], maize [6], *Populus* [31], barley [32], cucumber [33], and rice [34]. Recent studies have highlighted abiotic stress pathways in soybean, but only through meta-analysis [35, 36], and did not explore ML- with co-expression-based hub genes or biosignature identification. These methods have recently been applied to precisely identify biomarkers in diabetic nephropathy [37] and hepatocellular carcinoma [38], as well as to identify abiotic stress biosignatures in maize [33].

Gene duplication plays a crucial role in evolution, acting as a key driving force for the expansion of multigene families and generating new genes that contribute to ecological adaptation through natural selection [39]. Gene duplication contributes to the adaptation to adverse environmental conditions, such as different abiotic stresses in *Arabidopsis* and high salinity in poplar [40, 41]. The comparative meta-analysis of gene expression profiles in soybean across highly correlated abiotic stresses (heat, drought, and salinity), the identification of common stress-responsive genes with evolutionary significance, such as duplications, and the exploration of abiotic stress biosignatures through co-expression and an ML-based feature selection approach, have not yet been explored.

The current study utilized transcriptomic data to examine transcriptional-level gene regulation and to identify common stress-responsive genes (meta-DEGs) across heat, drought, and salt stress in soybean. The orthologous and paralogous relationships of meta-DEGs, along with their evolutionary patterns and divergence periods, were also explored. The abiotic stress biosignatures are identified and validated using artificial intelligence (AI), providing valuable insights into the use of AI in plant breeding and a solid basis for functional genomic approaches to improve crops through genome-assisted breeding.

## Materials and methods

### RNA-seq datasets

Transcriptome datasets from salt, drought, and heat stress-treated *G. max* plants were used in this analysis to identify gene-expression dynamics and abiotic-stress marker genes for stress-tolerant soybean breeding. Raw RNA-seq data from salt, drought, and heat stress-treated samples were retrieved from the NCBI SRA database accessed in May 2025. The meta dataset comprises 192 RNA-seq samples used for further analysis and is detailed in Table S1. Subsequently, the RNA-seq datasets were segregated into salt, drought, and heat libraries. A comparison was then conducted between the control and each stress condition (salt, drought, and heat) to evaluate gene expression changes induced by each stress factor.

### Transcriptome assembly

After conducting the FastQC check (v 0.12.0), the raw reads were processed to remove adaptor sequences, rRNA, and low-quality reads with fastp v 0.23.2 [42]. The resulting high-quality RNA-seq reads were then mapped to the soybean reference genome Wm82.a4.v1 [43], using HISAT2 v2.2.1 [44]. Subsequently, the aligned reads were assembled into transcripts with StringTie v 2.2.0 [45].

### Analysis of differential gene expression

Gene expression levels were calculated with StringTie to derive raw read counts for further analysis. To minimize bias in the diverse transcriptome data gathered from various bio-projects, batch factor normalization was performed on the read counts for salt, drought, and heat samples using the “RUVSeq” R package [46]. Because a universal set of housekeeping genes is unavailable, the RUVr method was used on the metadata sets. The number of unwanted factors (k) was chosen as 2, as this provided the best balance between sample clustering and reliable expression results. PCA was conducted both before and after normalisation of normal, salt, drought, and heat samples to examine the clustering pattern and the effectiveness of batch correction.

The batch-corrected read counts were subsequently normalized by the TMM (Trimmed Mean of M-values) normalization method [47] to derive CPM (Counts Per Million) reads for direct comparison across samples.The differential expression analysis was conducted using EdgeR version 4.6.3 [48], which employed a negative binomial distribution to model raw read count data. The data were subsequently adjusted for multiple testing with the Benjamini and Hochberg method [49]. Genes with a log2 fold change ≥ |±1| and a *p-*adj (FDR) ≤ 0.05 were considered as differentially expressed (DEGs).

### Enrichment analysis of the DEGs

Gene ontology (GO) enrichment analyses of common DEGs were performed using AgriGO v2.0 and DAVID [50, 51]. The common DEGs were initially mapped to their *Arabidopsis* homologs through sequence similarity search. The enrichment analysis was performed using DAVID and AgriGO, with entire *Arabidopsis* genome as the background gene set. The enrichment analysis of GO terms was conducted using FDR-adjusted *p*-values ≤ 0.05 with the Benjamini-Hochberg method. The KEGG tool (https://www.genome.jp/tools/kaas/) was used to identify the biological pathways in which the DEGs are involved. The Benjamini-Hochberg method was employed to identify enriched pathways at a False Discovery Rate (FDR) ≤ 0.05.

The Mercator tool was utilized for MapMan annotation of the identified DEGs, while the MapMan tool visualized the fold changes in expression levels under different stresses. Enrichment analyses of the MapMan pathways were performed using Wilcoxon Rank-Sum Test, with *p*-values adjusted using the Benjamini–Hochberg correction [52, 53].

### Analysis of the evolutionary relationships of the common DEGs

MCScanX was employed to find orthologous and paralogous gene pairs among DEGs from three stress conditions through an all-against-all BLASTP search (e-value < 1 × 10^-5^) [54]. The “duplicate_gene_classifier” module in MCScanX was used to identify duplicated genes. The genes with codes 1, 2, 3, and 4 represent dispersed (duplicated genes located in non-collinear genomic regions), proximal (duplicated genes located on the same chromosome but separated by a small number of intervening genes < 20), tandem (duplicated genes located adjacent to each other on the same chromosome), and WGD/segmental (duplicated gene pairs located within collinear genomic blocks) duplications, respectively. The paralogous and orthologous gene pairs of the common DEGs (meta-DEGs) were subsequently visualized using a Circos plot [55]. The rates of Ka and Ks substitutions were calculated using PAL2NAL program [56]. Gene pairs exhibiting high levels of synonymous substitution saturation (pS ≥ 0.75) were excluded from subsequent analyses because substantial divergence may yield unreliable Ks estimates and could bias the interpretation of divergence times and evidence of selection pressure. The divergence time, measured in million years ago (MYA), was estimated using the formula: T = Ks/2λ, where T denotes divergence time, and λ represents the constant rate of synonymous substitutions per nucleotide per year (1.5 × 10^-8^ for dicotyledonous plants) [57]. The selection pressure on each duplicated gene pair was evaluated using the Ka/Ks ratio, where Ka/Ks > 1 (positive selection), Ka/Ks < 1 (purifying selection), and Ka/Ks = 1 (neutral selection). In soybean, Ks values of 0.06 to 0.39 were considered recent duplication, and 0.40 to 0.80 were considered early legume duplication.

### Analysis of the co-expression network and identification of the hub genes 

WGCNA is a method that integrates gene expression data into co-expression modules, which allows the identification of complex relationships among modules and their correlations with various traits [58]. The normalized expression values (logCPM) of DEGs from salt, drought, and heat samples were used to construct a correlation network by using the WGCNA package for identifying gene co-expression modules exhibiting similar expression patterns. An adjacency matrix was created using a soft threshold of 8, as it was the lowest value that achieved a high fit to the scale-free topology model (*signed* R² > 0.9) while maintaining an acceptable mean connectivity, indicating that the resulting network achieved a scale-free topology. The “DynamicTreeCut” module was used for hierarchical clustering with module size 50, TOMType- unsigned, and a merging cutoff of 0.25.

The relationship between co-expression modules and abiotic stress traits such as salt, drought, and heat was derived from module-trait relationships and evaluated using Pearson correlation tests, with a *p*-value < 0.05 considered significant. Gene significance (GS) denotes the relationship between gene expression and different types of samples, where module membership (MM) correlates gene expression with the module eigengene (*signedKME*), employed to identify co-expression modules. The MM function with a cutoff > 0.8, indicating highly connected genes within the module, and the GS function with a cutoff > 0.2, indicating highly significant genes, were used to identify hub genes [59, 60]. The networks of gene co-expression were visualized using Gephi-0.10.1 software.

### Feature selection approaches for DEGs

Attribute weighting methods were used to select relevant features from 14,503 DEGs identified in the abiotic samples. The normalized expression value (logCPM) was used to identify key features across 175 samples. Six feature selection algorithms—Information Gain, Chi-squared, Uncertainty, Relief, Information Gain Ratio, and SVM—were applied to our datasets using the Python package Scikit-learn v1.2.4. The weights of each model were normalized between 0 and 1, with 1 indicating the highest importance. The abiotic stress signature genes were selected based on attribute weights, using a cutoff of a sum of weights > 4 [28, 61].

### Machine learning based pattern discovery model development for abiotic stress

Gene expression values (logCPM) for feature-selected genes were used to predict abiotic stresses, including salt, drought, and heat. All datasets were randomly split, with 80% allocated to training and 20% to testing. Eight machine learning models were applied for pattern discovery (Table 2). To evaluate models performance, all models were optimized and validated using 10-fold cross-validation with Python v 3.11.5 using Scikit-learn v1.2.4 [61, 62].

### Deep learning-based validation of stress-responsive genes

The abiotic stress-responsive hub genes identified through WGCNA and feature selection were validated using a Multi-Layer Perceptron (MLP) based deep neural network (DNN) model. All differentially expressed genes (DEGs) from three stresses were randomly split into 70% for training and 30% for testing. The model was trained with ‘epoch: 40’, ‘random state: 32’, three hidden layers with 64, 32, and 16 neurons, and dropout layers (0.3, 0.3, and 0.2) applied to reduce overfitting, using the ‘relu’ activation function. DNN model was optimized with the Adam optimizer at a learning rate of 0.001, and the model was validated via 5-fold cross-validation. To evaluate the model’s convergence and stability during training, the accuracy and loss curves are also generated. Additionally, an external validation set, consisting of a single bioproject for each stress condition (PRJNA551959 for salt stress, PRJNA641330 for drought stress, and PRJNA880914 for heat stress), was used to evaluate the deep learning (DL) model’s performance.

### Statistical analysis

All statistical analyses in our study were conducted using the R environment (v4.5.0) and Python (v3.10) to assess the correlation between genes of salt, drought, and heat stress samples. The performance of each model was evaluated using AUC (Area Under the Curve), accuracy, precision, lift, F-measure, sensitivity, and specificity. Furthermore, the AUC values of each model are presented as ROC curves.

## Results

### Analyses of abiotic stress transcriptome

To gain a comprehensive understanding of the dynamic responses of soybean gene expression under abiotic stress, high-throughput RNA-seq datasets from previously reported transcriptomes of salt, drought, and heat treated samples were incorporated. Consequently, comprehensive metadata sets were created featuring three stress libraries, salt, drought and heat, to discern high-confidence genes associated with abiotic stresses.

Approximately 545.49 GB of raw reads, comprising 1.026 trillion bases, were generated from normal healthy plants and from libraries for salt, drought, and heat using paired-end Illumina sequencing. After eliminating adapter sequences and low-quality reads, the data were refined to 986.1 billion clean reads. Following mapping to the *Glycine max* reference genome (Wm82.a4.v1) [43] an average alignment percentage of 89.51% was obtained. The mapping data were processed to identify DEGs using a custom pipeline [63].

### Differential expression of genes in response to abiotic stress

**Fig. 1:**
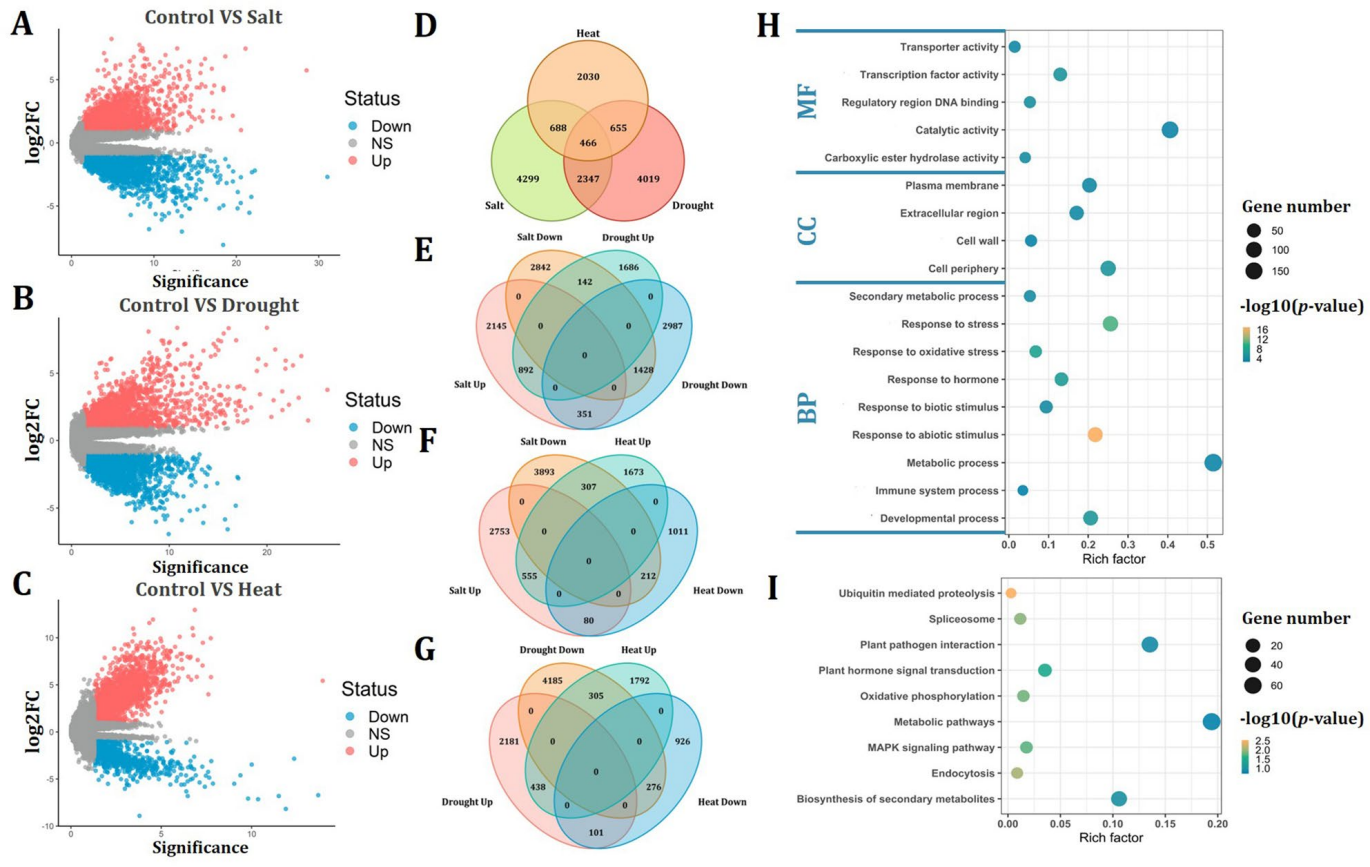
Functional annotation of the differentially expressed genes (DEGs) in salt, drought, and heat. **A-C**, Volcano plot of the up- and down-regulated differentially expressed genes in salt, drought, and heat stress against control samples. **D**, Venn diagram representing unique and common DEGs between salt, drought, and heat. **E-G**, Venn diagram of up- and down-regulated shared genes between salt vs. drought, salt vs. heat, and drought vs. heat. **H**, Gene ontology analysis of common DEGs categorized in terms of biological processes, cellular components, and molecular functions. **I**, Analysis of the enriched pathways responsive to the common DEGs

To remove unwanted variation (RUV), normalization was applied to the metadata set. The PCA plots show a reduction in batch-related variation and improved clustering of biologically similar samples after normalization (Figure S1). To investigate differential gene expression (DEG) patterns under salt, drought, and heat stress, relative gene expression levels were compared between each library and healthy control plants. In total, 7800 (3388 up and 4412 down), 7487 (2721 up and 4766 down) and 3839 (2536 up and 1303 down) -regulated genes were identified as DEGs in salt, drought and heat (log2FC ≤ − 1 or ≥ 1, FDR < 0.05), respectively, as depicted in Fig. 1A-C (Supplementary Table 2). Amongst the total DEGs, 4299 DEGs were unique to salt, 4019 DEGs were unique to drought, and 2030 were unique to heat stress, while 466 genes were commonly regulated in all three libraries. A total of 2347 DEGs are common to salt and drought stress, and 688 DEGs are common to salt and heat stress, respectively, whereas 655 DEGs are co-expressed in both drought stress and heat stress (Fig. 1D). The distinct patterns of up- and down-regulation among DEGs under salt, drought, and heat stress were shown in a Venn diagram (Fig. 1E-G).

### Functional enrichment analysis

To clarify potential roles, GO enrichment was performed on the common DEGs across the three stresses. The present study revealed statistically significant enrichments (*p* < 0.05), indicating the diverse roles of the DEGs in the plant stress-responsive pathways. Functional enrichments were classified into three distinct categories, namely BP (biological process), MF (molecular function), and CC (cellular compartment), based on their GO annotation. Enrichment analyses of the common DEGs across the three libraries (466 genes) identified 102 BPs, 10 MFs, and 10 CCs. Overall, the most significantly enriched GO terms in the biological process (BP) category included response to abiotic stimulus (74 genes), response to stress (87), response to oxidative stress (23), secondary metabolic process (18), and metabolic process (175). The enriched term from molecular function (MF) includes transcription factor activity (44 genes), transporter activity (18), and catalytic activity (138). The enriched term from cellular component (CC) includes cell periphery (85), plasma membrane (69), extracellular region (58), and cell wall (19) (Fig. 1H).

The KEGG pathway analysis of DEGs under salt, drought, and heat stress revealed the participation of 466 enzymes across 74 distinct biological pathways. Together, the most significantly enriched pathways encompassed metabolic pathways (66 enzymes), ubiquitin mediated proteolysis (10), endocytosis (13), spliceosome (26), oxidative phosphorylation (17), MAPK signaling pathway (28), plant hormone signal transduction (25), biosynthesis of secondary metabolites (36), plant pathogen interaction (46), and metabolic pathways (66) (Fig. 1I). These findings underscore the predominant association of the DEGs with metabolic pathways, secondary metabolite biosynthesis, plant pathogen interaction, oxidative phosphorylation, and MAPK signaling pathway upon onset of stresses from normal condition.

**Fig. 2:**
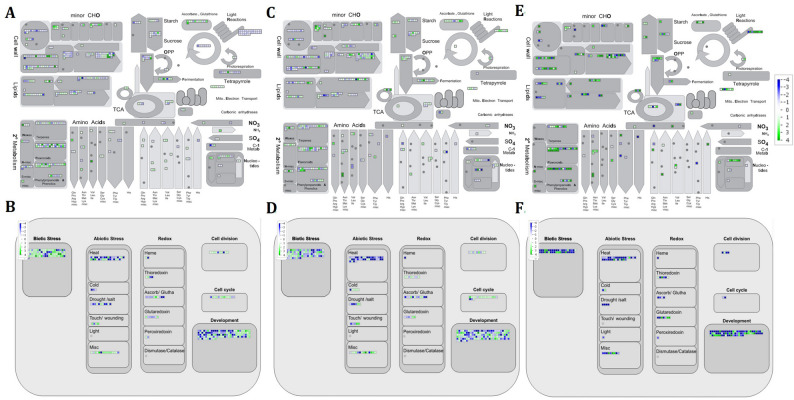
MapMan visualization of stress-responsive DEGs under stress conditions. Green represents up-regulated genes, and blue represents down-regulated genes. Differential expression of metabolic genes in soybean under salt (**A**), drought (**C**) and heat (**E**) stress. Participation of DEGs in various biological processes under salt (**B**), drought(**D**), and heat (**F**) stress

Analyses of the abiotic-stress (salt, drought, and heat)-induced transcriptomes revealed the physiological changes triggered by these stresses and mapped a significant portion of genes involved in metabolic pathways, which were highly enriched and identified through the MapMan pathway analysis (Fig. 2) (Supplementary Table 3). Genes involved in various components of the photosynthetic machinery showed widespread changes in their transcriptional regulation. The genes encoding transcripts for primary metabolism and the photosynthetic apparatus were repressed under stress conditions, whereas transcripts related to secondary metabolism were largely induced (Fig. 2A, C, E). These results suggest that alterations in metabolic gene expression under stress play a significant role in combating stress. The participation of DEGs under salt, drought, and heat stress in various biological processes, including abiotic stress, biotic stress, oxidative stress, cell division, cell cycle, and development, is shown in Fig. 2B, D, and **F**, and the adjusted *p*-values associated with the enriched MapMan pathways are available in Supplementary Table 3.

### Comparative analysis of stress-responsive genes

**Fig. 3:**
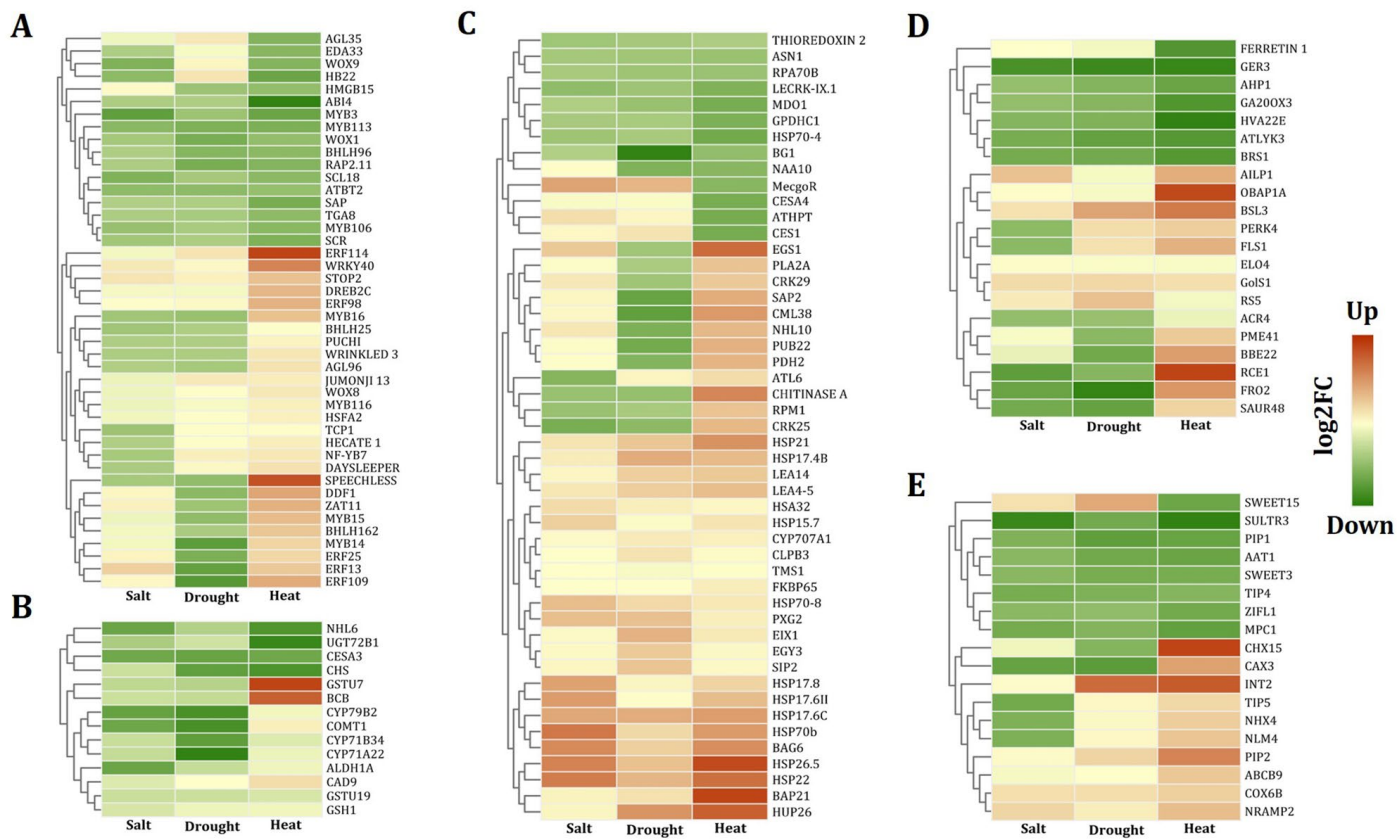
Expression analysis of differentially expressed genes related to. **A**, transcription factor. **B**, secondary metabolism. **C**, stress response. **D**, hormone signal. **E**, transporter respectively. Gene expression levels were visualized as heatmaps generated from the proportions of log2FC (Fold Change) values, with brown (upregulated) and green (downregulated) indicating expression in the salt, drought, and heat libraries, respectively

To explore the gene expression dynamics of abiotic stresses in soybean, stress-specific (salt, drought, and heat) expression profiles were evaluated, and RNA-Seq-derived fold-change values for each stress were computed for the DEGs. Gene regulatory networks govern nearly all aspects of plant stress responses, where transcription factors (TFs) are a crucial element of these networks. Previous reports highlight that transcription factors play a key role in plant responses to abiotic stresses [64]. The GO profile provides evidence that TF expression levels change significantly from normal to stress conditions, and that 44 TFs are co-expressed under salt, drought, and heat. These genes belong to the 17 TF family, including some members associated with stress tolerance. The most prominent members include the AP2ERF, MYB, bHLH, WRKY, and HSF transcription factor families, which have previously been reported to be involved in plant stress-responsive pathways [45, 48, 51]. Additionally, some plant development-related TFs, such as GRAS, MADS-box, and HB-WOX, are also differentially expressed across all three stresses. The three AP2/ERF family TFs, namely *ERF114*, *DREB2C*, and *ERF98*, are highly upregulated across all three stresses, whereas other ERF family members, such as *ERF25*, *ERF13*, and *ERF109*, are upregulated only under salt and heat stress, which have previously been reported to be involved in plant abiotic stress tolerance [65–67]. According to previous reports, overexpression of *WRKY40*, *STOP2*, *WOX8*, and *HSFA2* confers tolerance to various stress types [68–71]. The negative regulators of plant stress tolerance TFs, such as *ABI4* and *ATBT*, are highly downregulated across all three stresses [72, 73]. Elucidating the gene expression patterns of these TFs under stress will improve our understanding of plant stress-tolerance mechanisms (Fig. 3A).

Plants produce various secondary metabolites that primarily protect them from various stresses [74]. KEGG and MapMan provide substantial evidence that several secondary-metabolite biosynthesis genes were differentially expressed under stress conditions. The oxidative stress response enzymes glutathione S-transferase (*GSTU7* and *GSTU19*) and γ-glutamylcysteine synthetase (*GSH1*) are highly expressed under stress conditions. Cesarino (2019) reported that lignification helps plants combat stress [75]. The two lignin biosynthesis genes, Blue Copper-Binding protein (*BCB*) and Cinnamyl Alcohol Dehydrogenase 9 (*CAD9*), were upregulated under stress conditions. During salinity and heat conditions, two Cytochrome P450 enzymes, *CYP71B34* and *CYP71A22*, are highly expressed (Fig. 3B).

Exploring the expression profiles of stress-related genes revealed that the 11 Heat Shock Proteins (HSPs), two Late Embryogenesis Abundant (LEAs), and one Bcl-2-associated athanogene (*BAG6*) gene were highly expressed across all three stress conditions. These genes act as molecular chaperones to prevent protein misfolding during stress [76]. *HUP26* and *EGY3* are also highly expressed under all three stresses and have previously been reported to be involved in oxidative stress tolerance under heat and salt stress [77, 78] (Fig. 3C).

Analysis of the DEGs also revealed that plant hormone response and transporter family genes are highly differentially expressed under stress conditions (Fig. 3D, E) (Supplementary Table 4). Plant hormone signaling and transporter family proteins are crucial components of plant stress regulatory networks [79–81].

### Evolutionary relationship analysis of the meta DEGs

**Fig. 4:**
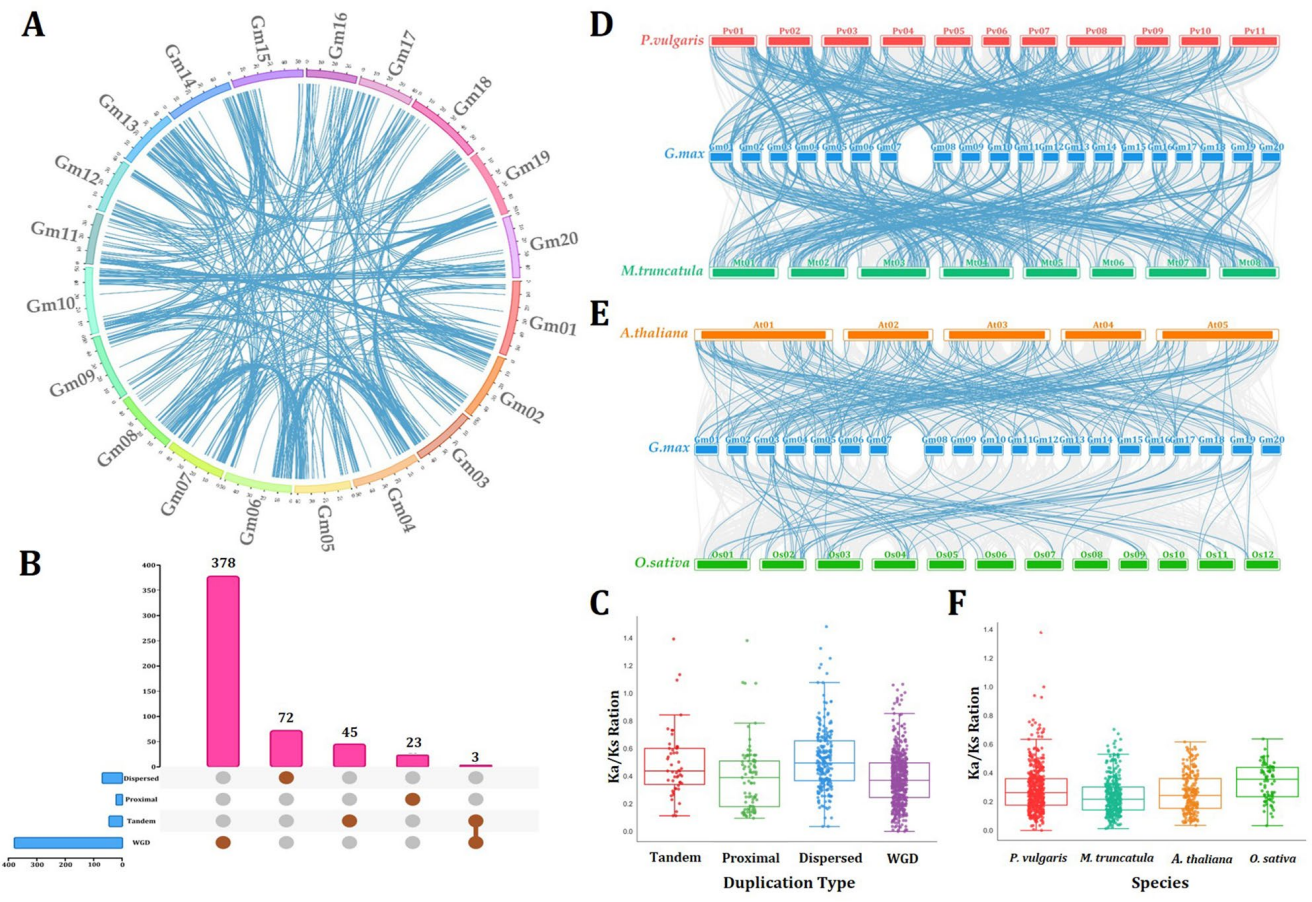
Paralogous and orthologous analysis of the meta-DEGs. **A**, Distribution of the paralogous genes across the twenty soybean chromosomes. **B**, Different types of duplication events across meta-DEGs. **C** Ka/Ks ratio of the different duplication events. **D**-**E**, Orthologous relationship of meta-DEGs present across different species. **F**, Ka/Ks ratio of the orthologs

Gene duplication contributes to the expansion of gene families. To investigate the occurrence of different types of gene duplication patterns (viz. dispersed, proximal, tandem and segmental or WGD), the common abiotic stress-responsive DEGs were analyzed. Among the 466 stress-responsive genes, 63.22%, 15.91%, 9.24%, and 5.16% were involved in segmental (WGD), dispersed, tandem, and proximal duplication, respectively (Fig. 4A, B) (Supplementary Table 5). All meta-DEGs (466 genes) comprise 907 duplicate gene pairs, of which 18 pairs are under positive selection (Ka/Ks > 1), one pair is under neutral selection (Ka/Ks = 1), and the rest are under purifying selection (Ka/Ks < 1) (Fig. 4C). The divergence time of the stress-responsive gene pairs was estimated to have occurred between approximately 0.6 and 74 Mya.

To assess syntenic relationships between soybean abiotic-stress-responsive genes and *Phaseolus vulgaris*, *Medicago truncatula*, *Arabidopsis thaliana*, and *Oryza sativa*, comparative genomic analysis was conducted to identify orthologous gene pairs (Fig. 4D, E). There were 298, 262, 140, and 56 genes that were orthologs of *Phaseolus vulgaris*, *Medicago truncatula*, *Arabidopsis thaliana*, and *Oryza sativa*, respectively. These genes form 460, 365, 212, and 75 syntenic pairs, respectively, in each species. A total of 39 soybean stress-responsive genes are orthologs across all four species. Around 82 genes are conserved in *Phaseolus vulgaris*, *Medicago truncatula*, and *Arabidopsis thaliana*, whereas 112 genes are conserved only in *Phaseolus vulgaris* and *Medicago truncatula*. The greater number of orthologous gene pairs of soybean with *Phaseolus vulgaris* and *Medicago truncatula* indicates genetic relatedness between these legume species over evolutionary time (Supplementary Table 6). The Ka/Ks distribution shows that all orthologous pairs are under purifying selection, except two pairs in *Phaseolus vulgaris*, one of which is under positive selection and the other under neutral selection **(**Fig. 4F**)**.

### Co-expression network analysis and hub genes identification

**Fig. 5:**
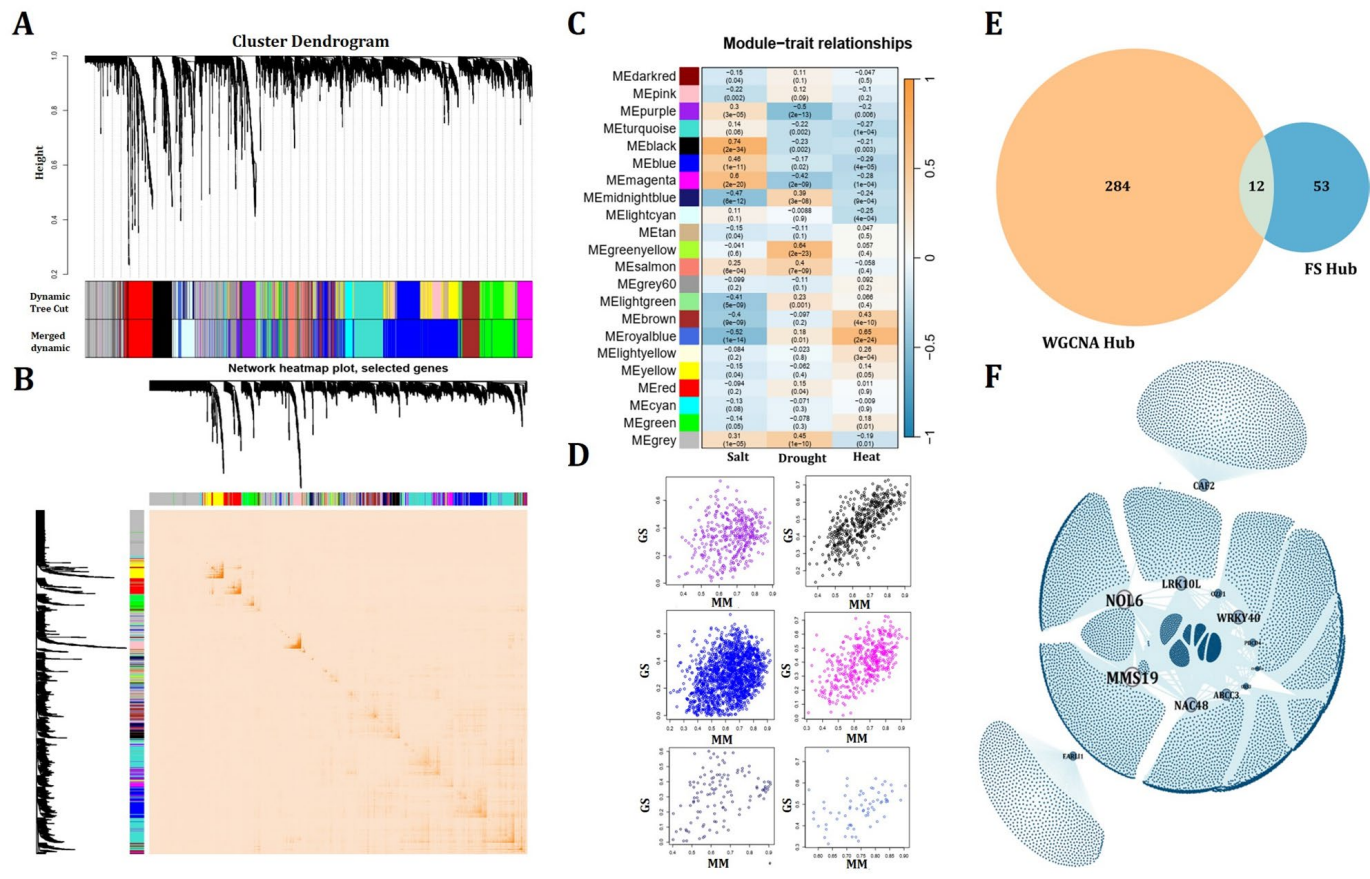
Co-expression network analysis of differentially expressed genes. **A**, Cluster dendrogram of the co-expressed modules is denoted by a colour bar. **B**,** C**o-expression gene interaction based on TOM dissimilarity and the cluster dendrogram. **C**, Heatmap of the correlation between salt, drought, and heat libraries with module eigengenes. Orange represents positive correlation, and blue represents negative correlation. The *P*-values are stated in the brackets. **D**, Gene significance and module membership correlation scatterplot of the significant modules. **E**, The venn diagram represents the hub genes derived from co-expression and ML-based feature selection analysis. **F**, WGCNA-based co-expression network analysis of the twelve biosignatures

To explore the potential biological roles of genes involved in abiotic-stress-responsive pathways, a co-expression network was constructed from DEGs associated with salt, drought, and heat stress. Weighted gene co-expression network analysis (WGCNA) was conducted, identifying 21 co-expressed modules and one unclustered module (MEgrey), as shown in the dendrograms (Fig. 5A, B), where different colours indicate distinct co-expressed modules. The Pearson correlation was calculated to assess relationships among modules that were significantly associated with all three stress samples (salt, drought, and heat stress). The six modules, such as “MEpurple”, “MEblack”, “MEblue”, “MEmagenta”, “MEmidnightblue” and “MEroyalblue”, show a significant association with all three traits and contain 410, 482, 1665, 463, 115 and 67 co-expressed genes, respectively (Fig. 5C). A higher module membership (MM) value indicates that a gene is highly representative of its module, while gene significance (GS) reflects the strength of association between gene expression and the trait of interest. We have identified a total of 296 hub genes within the correlated modules, based on the criteria MM > 0.8 and GS > 0.2 (Fig. 5D), ensuring that the identified genes have strong intramodular connectivity and biological relevance [59, 60]. The module “MEblue” contains the largest number of hub genes, 147, while the module “MEroyalblue” contains the smallest number of hub genes, 4, respectively (Supplementary Table 7).

Furthermore, to identify potential stress-responsive genes, six machine learning-based feature selection methods, such as Information Gain, Chi-squared, Uncertainty, Relief, Information Gain Ratio, and SVM, were applied to DEGs. They identified 65 genes with a total attribute-weighting score exceeding 4 in the abiotic stress-responsive transcriptomic signature (Supplementary Table 8). Twelve common hub genes were identified between WGCNA-based hub genes and attribute-weighted-based genes (Fig. 5E). Analysis of the co-expression network of the common hub genes shows that five genes, namely *MMS19*, *NOL6*, *NAC48*, *LRK10L*, and *WRKY40*, regulate more than two thousand abiotic stress gene signatures. Other genes, including *ABCC3*, *OZF1*, *PDCD4*, *OXS3*, *CAF2*, *CYP82C4*, and *EARLI1*, regulate 1797, 1299, 1131, 876, 650, 624 and 434, respectively (Fig. 5F).

### Performance of pattern discovery machine learning models in transcriptomic signatures

**Fig. 6:**
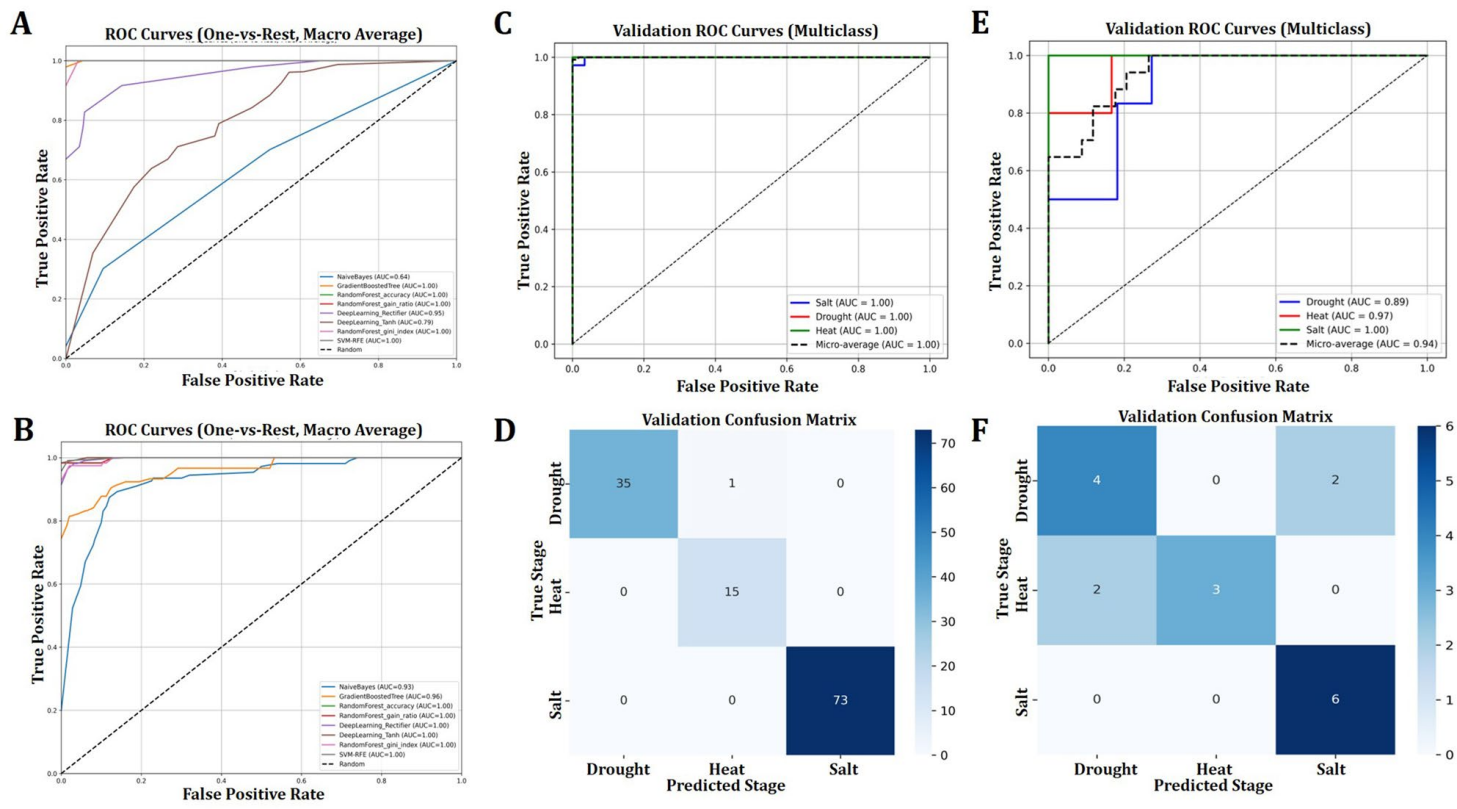
Biomarker validation by ML and DL approach. **A**, ROC curves and AUC scores comparing 8 machine learning models before feature selection. **B**, ROC curves and AUC scores comparing 8 machine learning models after feature selection. **C**, ROC curves and AUC scores for classifying salt, drought, and heat on total soybean genes using a deep neural network (DNN) model. **D**, Confusion matrix of the DNN model for the total soybean genes for salt, drought, and heat libraries. **E**, ROC curves and AUC scores for classifying salt, drought, and heat on external sets using a deep neural network (DNN) model. **F**, Confusion matrix of the DNN model for the external validation sets for salt, drought, and heat libraries

To investigate the transcriptomic signature (DEGs) for distinguishing among salt, drought, and heat, eight pattern discovery machine learning models were employed using 14,503 signature gene expressions as features, and their predictive performance was compared. The accuracy of each model is at least 60%, except for DeepLearning_Tanh, which achieves 40%. The SVM-REF model achieves the highest accuracy, at 97.11% (Table 1). The macro-average ROC curve for all models is shown in Fig. 6A.

**Table 1:** Performance comparison of machine learning models before feature selection

Models	Accuracy (%)	AUC	Precision (%)	F1-Score	Sensitivity (%)	Specificity (%)
NaiveBayes	60	0.637164	57.19697	41.079509	42.857143	84.575569
GradientBoostedTree	97.142857	0.999094	93.75	95.341615	97.916667	99.21875
RandomForest_accuracy	97.142857	0.997396	98.076923	94	91.666667	98.913043
RandomForest_gain_ratio	97.142857	0.997396	98.076923	94	91.666667	98.913043
DeepLearning_Rectifier	60	0.937061	49.09188	53.280423	59.52381	84.777756
DeepLearning_Tanh	40	0.780586	10	14.285714	25	75
RandomForest_gini_index	97.142857	0.997396	98.333333	94.137931	91.666667	98.809524
SVM-RFE	97.116883	0.968043	97.25316	94.702387	93.691571	92.921371

To further investigate the importance of the attribute-weighting-derived transcriptomic signature in distinguishing among samples from salt, drought, and heat, eight machine learning models were applied to 65 signature genes as features, and their predictive performance was assessed. It was observed that the accuracy of each model exceeded 70%. The RandomForest and GradientBoostedTree models attained the highest accuracy 98.7% (Table 2) after feature selection. The predictive performance of the salt, drought, and heat samples after feature selection for all models is shown in Fig. 6B.

**Table 2:** Performance comparison of machine learning models after feature selection

Models	Accuracy (%)	AUC	Precision (%)	F1-Score	Sensitivity (%)	Specificity (%)
NaiveBayes	83.116883	0.958043	85.25316	83.702387	82.691571	93.921371
GradientBoostedTree	98.701299	0.997849	99.107143	98.683386	98.333333	99.5
RandomForest_accuracy	98.701299	0.997849	99.107143	98.683386	98.333333	99.5
RandomForest_gain_ratio	96.103896	0.996804	96.22549	96.543183	97.222222	98.672715
DeepLearning_Rectifier	94.805195	0.998925	94.736842	95.058824	96.296296	98.387097
DeepLearning_Tanh	98.701299	0.997849	99.107143	98.683386	98.333333	99.5
RandomForest_gini_index	97.402597	0.997981	97.511574	97.829026	98.212005	99.096774
SVM-RFE	93.116883	0.958043	96.25316	93.702387	92.691571	96.921371

### Validation of the stress-responsive genes

Based on stress-responsive gene signatures identified from hub genes and feature selection, a multi-level classification-based deep neural network (DNN) model was developed. We used DEGs from salt, drought, and heat stressed samples as training sets to validate the 12 signature genes as inputs, with salt, drought, and heat as classification labels, and validated the model with 5-fold cross-validation, achieving an accuracy of 97.39% with a standard deviation of 5%. The model achieved an accuracy of 99.19%, sensitivity of 99%, and a precision of 98% on soybean whole-gene expression data, indicating robust performance under noisy gradients. The ROC curve and confusion matrix were also plotted to evaluate the prediction performance of our multi-class deep learning model (Fig. 6C, D). The 5-fold cross-validation loss curves (mean ± standard deviation) show that the model converges reliably and stably across various data splits. The tight confidence intervals suggest low variability and good reproducibility. Notably, validation loss remains consistently below training loss, which helps improve generalization, and there is no indication of overfitting (Figure S2). One BioProject for each stress sample was used for external validation to assess the importance of the 12 signature genes in predicting salt, drought, and heat stress. The DNN model achieved an accuracy of 76.47%, with sensitivities of 76% and a precision of 81% on external validation sets (Supplementary Table 9). The ROC curve and confusion matrix for the external validation sets were also plotted to assess the prediction performance of our multi-class deep learning model (Fig. 6E, F). These results demonstrate the potential of these genes as a signature for predicting stress conditions and highlight their roles in various abiotic stresses (salt, drought, and heat), aligning with our study’s goal of uncovering the intricate links among these stresses and their potential involvement in stress-tolerant plant breeding.

## Discussions

To cope with unfavourable environmental conditions, plants alter their gene expression profiles in response to different types of stress [82, 83]. Extreme weather conditions and climate change causes high temperature, leading to droughts and increased soil salinity [5]. Exploring the common hub genes responsive to various abiotic stresses enables the development of multi-stress-tolerant cultivars. Meta-analysis has recently gained attention for its potential to enhance the statistical power and generalizability of the study [25]. The gene expression dynamics under multiple stress conditions in soybean plants were analyzed to understand environmental responses. The availability of RNA-Seq data on soybean responses to various abiotic stresses, such as salt, drought, and heat, prompts us to conduct a meta-analysis to elucidate the common genetic factors underlying abiotic-stress responses and enable the breeding of multi-stress-tolerant soybean varieties. In this study, high-throughput transcriptome data of three stresses were analyzed to identify the transcriptional changes and transcript abundance during stress intervention.

The impact of abiotic stresses on soybean transcriptional regulation is substantial, with more than 14,500 genes differentially expressed across three stress conditions (salt, drought, and heat). Under salt and drought stress, nearly 14% of genes in the soybean genome were differentially expressed, while approximately 7% were affected under heat stress, indicating that salt and drought stress are more devastating for soybean plants. The MapMan analysis showed a significant impact on plant metabolism in stress-treated plants (Fig. 2), with an increased number of secondary metabolic genes, suggesting their potential in stress-tolerant pathways [74]. Furthermore, the 466 genes (nearly 0.9% genes of the soybean genome) that are commonly present across three stresses participate in pathways of oxidative stress, secondary metabolism, hormone signalling, and MAPK signalling, which have previously been reported to be linked to stress-resistant pathways (Fig. 1H, I**)** [9, 39, 74, 84].

Exploring insights into the common genes reveals that nearly 10% are transcription factors. According to previous data, TFs are involved in multiple stress-signaling pathways, resulting in the activation of the same sets of genes in response to different stresses [85]. The most prominent TFs observed in our analysis are AP2ERF, WRKY, and HSF, which are involved across three stresses and are reported to be involved in plant stress tolerance [65, 86, 87]. The AP2/ERF family TFs, namely *ERF114*, *DREB2C*, and *ERF98*, are highly upregulated across three stresses. The *ERF114* gene is involved in plant defence against fungal pathogens, whereas overexpression of *DREB2C* and *ERF98* enhances plant tolerance to heat and salt stress, respectively [88–90]. The WRKY and HSF family members, *WRKY40* and *HSFA2*, are also highly expressed across all three stress conditions. The overexpression of *WRKY40* confers enhanced tolerance to cold and drought stress, whereas *HSFA2* amplifies the expression of heat stress-responsive genes in *Arabidopsis* [91, 92]. Oxidative stress response genes, such as *GSTU7*, *GSTU19*, and *GSH1*, are also highly expressed across all stresses. Several studies reported that glutathione reduces the effects of abiotic stress-mediated damage in plants [93]. Other stress-tolerant genes, such as Heat Shock Proteins (HSPs) and Late Embryogenesis Abundant (LEAs), are also upregulated under stress conditions. The majority of previously reported stress-responsive genes in meta-DEGs showed similar expression patterns; based on these observations, we conclude that some genetic factors regulate other stress-responsive genes and may be useful for multi-abiotic stress-tolerant soybean breeding. Some research also supports our hypothesis that certain genes regulate multiple abiotic stresses; notable studies include those in *Arabidopsis thaliana* and *Marchantia polymorpha* [30, 94].

The evolution of gene families is driven by gene duplication events, which are considered prominent forces in the creation of novel gene functions [95]. Studying duplication events in soybean, which influence the expansion of stress-responsive genes, it was observed that more than 60% of genes are involved in whole genome duplication (WGD), suggesting that WGD is the major driving force for stress-responsive gene expansion. This aligns with previous reports indicating that more than 50% of soybean genes are involved in WGD [96]. Among the total whole-genome duplication pairs in stress-responsive genes, 44.25% are from the recent WGD in soybean, whereas 24.61% are from the early legume duplication, which aligns with previously reported soybean duplication data [43]. Purifying selection is the major driving force in the evolution of soybean stress-responsive genes, suggesting that sub-functionalization is the main fate of duplicated genes [97]. Additionally, 18 pairs of stress-responsive genes underwent neo-functionalization, suggesting that a small fraction of genes acquired new functions through positive selection. This result suggests that during soybean evolution, expansion of stress-responsive genes occurred multiple times, with transcriptionally active forms, and these genes are valuable resources for identifying abiotic stress-tolerant QTLs in soybean. The greater number of abiotic stress-responsive orthologous genes in *Phaseolus vulgaris*, *Medicago truncatula*, and *Arabidopsis* than in rice indicates their genetic relatedness and the presence of dicot-specific orthologs. The 39 orthologous genes are present across all four plants, indicating that some stress genes are conserved over evolutionary timescales and constitute the core stress-responsive genes. By contrast, 112 genes are present only in legumes, indicating that they contribute to lineage-specific adaptation under stress conditions.

Furthermore, 12 abiotic stress-responsive hub genes (biosignatures) were identified from a co-expression network using WGCNA and machine learning (ML)-based feature selection algorithms. WGCNA and machine learning have recently emerged as accurate methods for identifying hub genes, which act as master regulators of specific cellular responses [61, 98, 99].

The most prominent biosignatures in soybean abiotic stress include *WRKY40*, *NAC48*, *LRK10L*, *OZF1*, *PDCD4*, *OXS3* and *EARLI1*, which have previously been reported to be involved in various types of plant abiotic stress tolerance. In 2022, Lin et al. reported that overexpression of *WRKY40* in *Pyrus betulaefolia* plants confers high tolerance to salt stress by inducing the expression of the V-type H+-ATPase gene (*VHA-B1*), which plays a significant role in plant stress responses [68]. Additionally, overexpression of *WRKY40* in *Malus baccata* increases tolerance to drought and cold stress by scavenging ROS (reactive oxygen species) through the positive regulation of antioxidant enzyme expression [91]. Overexpression of *NAC6* in rice, the homolog of *NAC48*, confers tolerance to several abiotic stresses, including drought, cold, and high salinity by enhancing the expression of antioxidant enzymes like thioredoxin, peroxidase and lipoxygenase [100, 101]. The mutation in the *Arabidopsis* receptor-like protein kinase (*AtLRK10L*) confers reduced tolerance to drought stress, whereas overexpression results in an enhanced drought tolerant phenotype [102]. The high expression of oxidation-related zinc finger 1 (*OZF1*) and oxidative stress 3 [*OXS3*] in *Arabidopsis* enhances the plant’s resistance to oxidative stress [103, 104]. Overexpression of the *EARLI1* gene improves germinability under salt and low-temperature stress, whereas *PDCD4* improves tolerance to salt stress [105, 106]. Other important genes include *MMS19*, an iron-sulphur (Fe-S) cluster protein in rice that confers resistance to oxidative and heavy-metal stress, and ATP-binding cassette C (*ABCC*) in maize, which enhances resistance to salt and cold stress [107, 108]. Additionally, novel biosignatures such as Nucleolar protein 6 (*NOL6*), CRS2-associated factor 2 (*CAF2*), and the cytochrome P450 family protein *CYP82C4* have not previously been reported to be involved in plant stress responses. Pathak et al. (2025) reported that *NOL6* acts as a hub gene under abiotic stress conditions [36]. Further research is needed to understand the role of these novel biosignatures in soybean responses to stress. The presence of *WRKY40* in both common DEGs and biosignatures indicates that it may play a key regulatory role in coordinating various stress responses in soybean, which was not previously reported and could serve as a potential candidate for breeding soybean varieties with enhanced abiotic stress tolerance. Based on our findings, soybean abiotic stress biosignatures are associated with oxidative stress, which helps protect plants by scavenging ROS during stress conditions, thereby making oxidative stress a central feature of the stress response. Therefore, we conclude that reducing oxidative stress in any stress condition by regulating biosignatures would increase soybean plant tolerance to multiple abiotic stresses.

Additionally, the pattern discovery model highlighted the importance of machine-learning-based feature selection methods, achieving higher recognition accuracy than for DEGs under salt, drought and heat stress, elucidating the importance of feature selection in hub gene detection. Xie et al. (2024) developed a deep neural network (DNN) model to validate the identified efferocytosis-related biomarkers in lung adenocarcinoma [109]. We constructed a deep learning (DL) based deep neural network (DNN) to validate the significance of the twelve biosignatures. To substantiate the predictive power of these abiotic-stress-responsive gene features, a multi-class DNN was used with the biosignature as input and three abiotic stresses (salt, drought, and heat) as class labels. The model achieved 97.39% accuracy with 5-fold cross-validation and 76.47% accuracy on external validation sets. Both loss and accuracy curves (mean ± standard deviation) demonstrate smooth convergence with minimal variability between folds. The validation loss remains lower than the training loss, and the validation accuracy is higher than the training accuracy, likely due to the dropout regularization used during training. The tight confidence intervals in later epochs suggest consistent and reliable learning (Figure S2). There is no sign of overfitting, confirming the model’s strong ability to generalize. The results demonstrate that these genes have potential as biosignatures of abiotic stress and act as the most central genes, highlighting their role in multi-abiotic stress tolerance in soybean plant breeding.

In summary, our study provides evidence that some core genes are involved in multiple abiotic stresses, and understanding their evolutionary patterns, such as paralogous and orthologous relationships, is crucial for improving stress tolerance in legumes and other crops. Identifying stress-responsive biosignatures and evaluating their significance using AI-based methods, along with insights into their roles in stress responses, provides a valuable resource for future investigations in molecular breeding. This study provides a solid foundation for AI applications in plant breeding to improve soybeans and also contributes important links between gene expression data and functional genomics.

## Conclusion

In conclusion, RNA-Seq data reveal a comprehensive transcriptional-level gene regulation and identify common stress-responsive genes (meta-DEGs) across heat, drought, and salt stress in soybean. The GO and KEGG pathway enrichment analyses of the meta-DEGs provide clues to the mechanisms underlying soybean responses to abiotic stress. Expression analysis of the stress-responsive meta-DEGs revealed similar expression patterns across the three stress conditions, highlighting their potential to regulate other stress-responsive genes and suggesting their utility for multi-abiotic stress-tolerant soybean breeding. Segmental or WGD is the major driving force for stress-responsive gene family expansion in soybean. Furthermore, by utilizing machine-learning-based feature selection and a WGCNA-based co-expression network, abiotic-stress-responsive hub genes or biosignatures were identified and validated using an artificial intelligence (AI)- based deep neural network (DNN), paving the way towards multi-abiotic-stress-tolerant soybean through genome-assisted breeding.

## Supplementary Information

Below is the link to the electronic supplementary material.

Supplementary Material 1

Supplementary Material 2

## Data Availability

The original contributions presented in the study are included in the Supplementary Material. Further supporting data for the findings of this study are available from the corresponding authors on request.

## Abbreviations

DEG: Differentially Expressed Genes
PCA: Principal Component Analysis
CPM: Counts Per Million
GO: Gene Ontology
KEGG: Kyoto Encyclopedia of Genes and Genomes
WGD: Whole Genome Duplication
MYA: Million Years Ago
WGCNA: Weighted Gene Correlation Network Analysis
TOM: Topological Overlapping Matrix
GS: Gene Significance
MM: Module Membership
AI: Artificial Intelligence
FS: Feature Selection
ML: Machine Learning
DL: Deep Learning
DNN: Deep Neural Network
AUC: Area Under the Curve
