# Supplementary material for: Harnessing meta-analysis and artificial intelligence to reveal conserved regulatory biosignatures of abiotic stress in soybean
Source: Biol Direct. 2026 May 11;21:120. doi: 10.1186/s13062-026-00788-2 (PMC13334898; doi:10.1186/s13062-026-00788-2)
Supplement: Supplementary file 2 — Supplementary Material 2 [file 13062_2026_788_MOESM2_ESM.docx]

**Supplementary Figures**


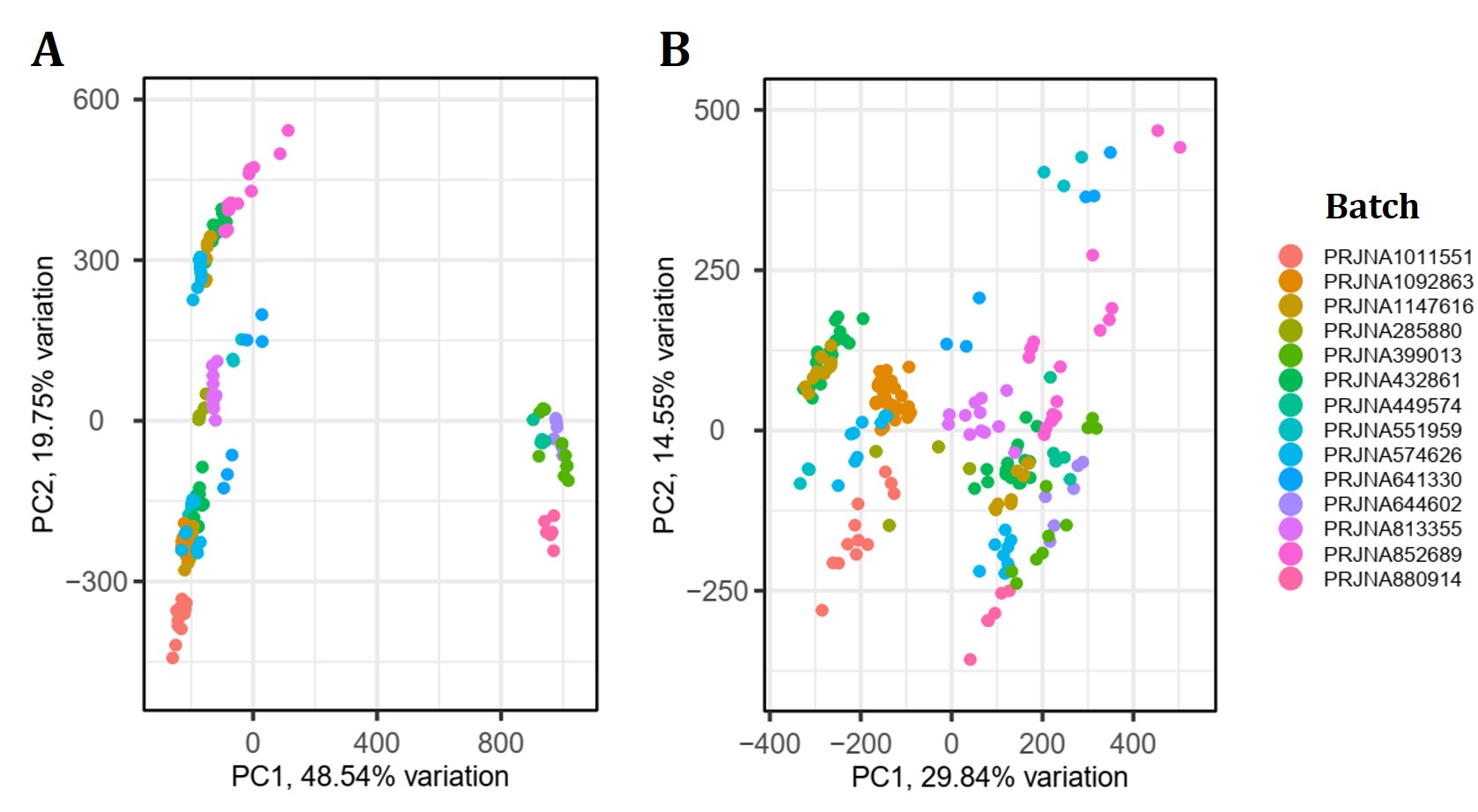


**Figure S1**: PCA analysis for the effectiveness of batch effect correction. (**A**) PCA before batch effect correction. (**B**) PCA after batch effect correction.


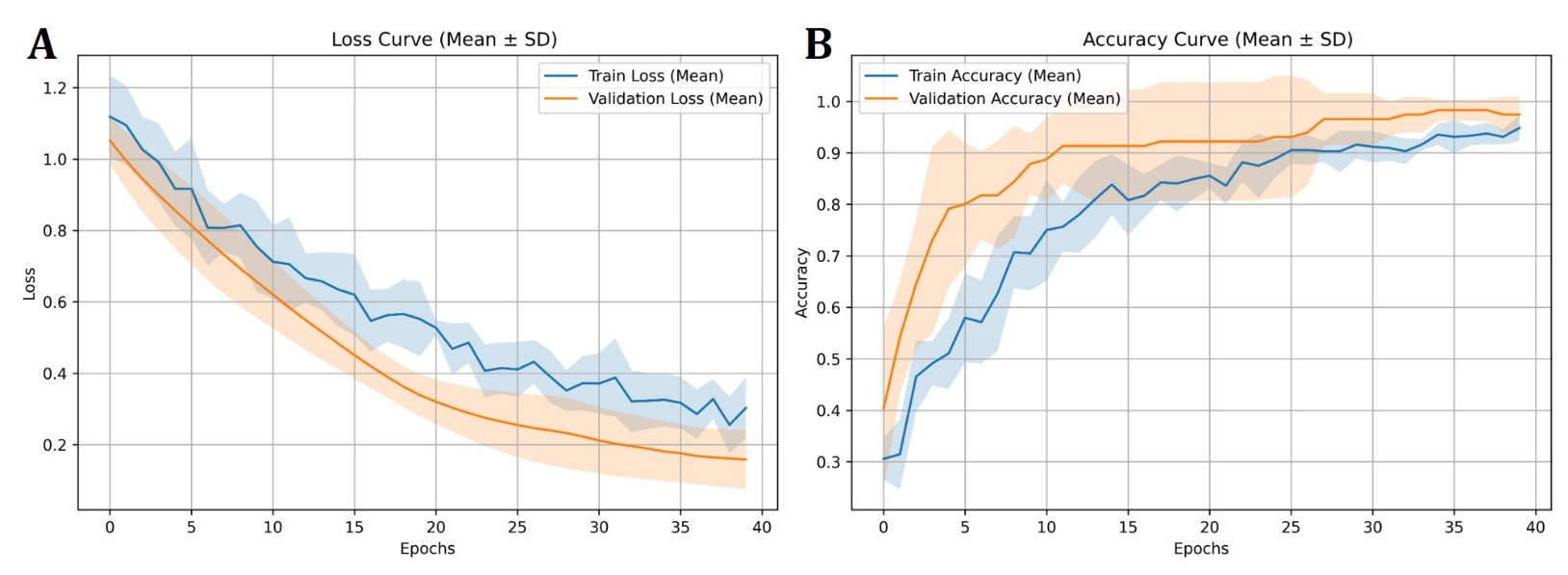


**Figure S2**: Performance evaluation of the deep learning model using 5-fold cross-validation. (**A**) Training and validation loss curves showing stable convergence and low inter-fold variability. (**B**) Training and validation accuracy curves demonstrating consistent improvement in classification performance with high reproducibility. The narrow confidence intervals indicate robustness of the model across different data splits.
